# Repetitive Sequence Distribution on *Saguinus*, *Leontocebus* and *Leontopithecus* Tamarins (Platyrrhine, Primates) by Mapping Telomeric (TTAGGG) Motifs and rDNA Loci

**DOI:** 10.3390/biology10090844

**Published:** 2021-08-30

**Authors:** Simona Ceraulo, Polina L. Perelman, Sofia Mazzoleni, Michail Rovatsos, Francesca Dumas

**Affiliations:** 1 Department of “Scienze e Tecnologie Biologiche, Chimiche e Farmaceutiche (STEBICEF)”, University of Palermo, 90100 Palermo, Italy; simona.ceraulo@community.unipa.it; 2 Institute of Molecular and Cellular Biology, SB RAS, 630090 Novosibirsk, Russia; perelmanp@mcb.nsc.ru; 3 Department of Ecology, Faculty of Science, Charles University, 12844 Prague, Czech Republic; sofia.mazzoleni@natur.cuni.cz (S.M.); michail.rovatsos@natur.cuni.cz (M.R.)

**Keywords:** heterochromatin, telomeric sequences, rDNA loci, tamarins, *Saguinus*, *Leontocebus*, *Leontopithecus*

## Abstract

**Simple Summary:**

Telomeric and rDNA sequence distribution on tamarins (New world monkeys, Primates) was analysed through molecular cytogenetics by fluorescence in situ hybridization. The mapping of Telomeric and rDNA probes on chromosomes was performed in order to clarify their localization and role in genome evolution. We found rDNA loci on the same homologs 19–22 on the analysed species with a different position in one of them named Leontopithecus rosalia, presumably as result of inversions. Other rDNA signals could be present on chromosome 16 and 17. On the last species, we found the classic telomeric sequence with exceptions while on the other species analysed, we found very amplified telomeric signals at the edge of chromosomes and some centromeric signals as exceptions, especially on chromosome pairs 16 and 17 as result of inversions of telomeric sequences or the presence of new acquired rDNA loci above them. The results obtained enable us to underline that the different chromosomal morphology between the species analysed could be due to inversions which dislocate the rDNA loci, the presence of new rDNA loci or the amplification of telomeric sequences. A comparative perspective with other data results obtained could be useful in order to better understand genome evolution.

**Abstract:**

Tamarins are a distinct group of small sized New World monkeys with complex phylogenetic relationships and poorly studied cytogenetic traits. In this study, we applied molecular cytogenetic analyses by fluorescence in situ hybridization with probes specific for telomeric sequences and ribosomal DNA loci after DAPI/CMA3 staining on metaphases from five tamarin species, namely *Leontocebus fuscicollis, Leontopithecus rosalia, Saguinus geoffroyi, Saguinus mystax* and *Saguinus oedipus*, with the aim to investigate the distribution of repetitive sequences and their possible role in genome evolution. Our analyses revealed that all five examined species show similar karyotypes, 2n = 46, which differ mainly in the morphology of chromosome pairs 16–17 and 19–22, due to the diverse distribution of rDNA loci, the amplification of telomeric-like sequences, the presence of heterochromatic blocks and/or putative chromosomal rearrangements, such as inversions. The differences in cytogenetic traits between species of tamarins are discussed in a comparative phylogenetic framework, and in addition to data from previous studies, we underline synapomorphies and apomorphisms that appeared during the diversification of this group of New World monkeys.

## 1. Introduction

Among New World monkeys, the subfamily Callitrichinae (Cebidae; Platyrrhini) represents one of the richest groups in terms of species and phenotypic variation. It consists of 48 currently recognised species [1] assigned to three tamarin genera (*Saguinu*s, *Leontocebus* and *Leontopithecus*) and four marmoset genera (*Callithrix*, *Cebuella, Callimico* and *Mico*). The classification and phylogenetic position of marmosets and tamarins have been quite extensively debated. The position of the genera *Leontopithecus* and *Saguinus* (tamarins) was controversial, with either *Saguinus* or *Leontopithecu*s considered as basal [2]; the most recent phylogenetic arrangements assign the genus *Saguinus* as the most basal lineage, followed by *Leontopithecus, Callimico, Callithrix, Mico* and *Cebuella* in agreement with the phyletic dwarfism hypothesis. This hypothesis proposes an evolutionary trend from large size ancestral forms to the smallest platyrrhine derived forms [3,4,5]. Despite the complexity of tamarin and marmoset phylogeny, classic cytogenetic investigations presented a stable scenario. The species of the genus *Saguinus* have rather similar G-banded karyotypes with differences in heterochromatin distribution, as revealed by a C-banding comparison. Indeed, many species of the genera *Saguinus*, *Leontopithecus* and *Leontocebus fuscicollis* share the same diploid number 2n = 46 but differ mainly by the C-banding pattern [6,7,8,9,10,11,12]; for example, *Saguinus midas* and *Leontopithecus rosalia* have similar chromosome morphologies, but they differ through a paracentric inversion and four pericentric inversions, as well as through the distribution and quantity of heterochromatin [11,12].

Comparative chromosomal painting between representative species of *Saguinus* and *Leontopithecus* did not reveal interchromosomal rearrangements [13,14,15], but intrachromosomal rearrangements have been hypothesised through cross-species comparison of conventional G-banding [11,12] and mapping of BACs (Bacterial Artificial Chromosomes) by FISH [16,17]. A large portion of the genome in Primates consists of repetitive DNA sequences, including tandem and dispersed satellite repeats [18]. The study of these repetitive elements offers interesting insights into karyotype evolution [18,19,20,21,22], especially for the distribution of ribosomal DNA (rDNA) loci and telomeric (TTAGGG)_n_ sequences. These elements have been successfully used as markers for comparative cytogenetic and phylogenetic studies [19,20,23,24,25,26].

The 45S rDNA regions, also known as the Nucleolus Organizer Regions (NORs), comprising the 5.8S, 18S and 28S ribosomal subunits, can be identified either by silver staining or, more accurately, by rDNA-FISH, which permits researchers to identify both active and inactive NORs. The NORs have been identified in many primates and the analysis of their topology, and can be informative for the karyotype evolution and the phylogenetic relations between species [20,22]. The variation in number and topology of rDNA loci has been shown at the inter- and also at the intra-species level, most commonly explained as a consequence of chromosomal rearrangements, transposition events or ectopic recombination through association of rDNA loci with other chromosomal segments occurred during meiotic division [22,27,28]. The topology of rDNA loci has been identified in a few species of the genus *Saguinus* by classical and molecular methods [7,11,12,21,29], in *Leontopithecus rosalia* through silver staining [11] and in *Leontopithecus chrysomelas* by rDNA-FISH [14].

Telomeric (TTAGGG)_n_ sequences in long tandem arrays characterise the terminal regions of chromosomes in the vast majority of animal taxa [30]. Nevertheless, these sequences have also been found at centromeric, pericentromeric and/or intermediate positions between the centromere and telomeres [19,23,31]. When localised outside the terminal positions of the chromosomes, these sequences are referred as Interstitial Telomeric Sequences (ITSs) or Interstitial Telomeric Repeats (ITRs). ITSs have been often related to chromosomal rearrangements such as fusion, fission and inversion, and to mechanisms of genome reorganisation, such as double DNA strand break repair [23]. Furthermore, ITSs are often correlated with heterochromatinisation, especially at centromeric positions (het-ITSs) [19,20]. Among tamarins, the distribution of telomeric (TTAGGG) sequences has been studied in *Saguinus oedipus* [19], *S. midas* and *S. bicolor* [29]. 

The aim of the present study is to expand our knowledge on the characterisation of the genome of tamarins by applying both classical and molecular cytogenetic approaches to closely related species, namely *Leontocebus fuscicollis, Leontopithecus rosalia, Saguinus geoffroyi, S. mystax* and *S. oedipus*, and to explore the distribution of heterochromatin and repetitive sequences in their karyotypes and their possible role as cytogenetic markers that are useful in evolutionary and phylogenetic comparisons.

## 2. Materials and Methods

### 2.1. Sampling Material

Following standard protocol [32], metaphase chromosome spreads were obtained from primary fibroblast cell cultures of *Leontocebus fuscicollis, Leontopithecus rosalia, Saguinus geoffroyi, S. mystax* and *S. oedipus* (Table 1). 

### 2.2. Karyotype Analysis

The species were karyotyped by 4′,6-diamidino-2-phenylindole (DAPI) inverted banding: after DAPI staining of metaphases chromosomes, pictures were analysed through the software Adobe Photoshop to get the inverted DAPI staining. The karyotypes matched published Giemsa Banding data for *S. geoffroyi* [7], *S. oedipus* [7,13], *S. mystax, Leontocebus fuscicollis* [9,11,12] and *Leontopithecus rosalia* [12,13]. Chromosomes were classified according to the nomenclature proposed by Levan et al. [33]. The pattern of heterochromatin distribution was analysed with CG-specific chromomycin A3 (CMA3) and AT-specific DAPI sequential staining with the aim to respectively detect GC/AT rich regions following the protocol presented in Lemskaya et al. [34] and Scardino et al. [26].

### 2.3. Fluorescence In Situ Hybridisation (FISH) and C-Banding

The distribution of the telomeric motifs was analysed by FISH with the PNA oligonucleotide probe (Panagene Cambridge Research Biochemical) FITC labelled. Hybridisation was performed following the protocols purchased by Panagene, adjusting stringency conditions [19]. We performed our FISH experiment both at high and low stringency in order to better detect both terminal and interstitial signals [19]. High stringency detection was performed with high temperatures at 70 °C and a low saline concentrate buffer, while low stringency was performed at lower temperatures at 37 °C and with a high saline buffer concentration. 

The probe for the rDNA sequence was prepared from a plasmid (pDmr.a 51#1) with a 11.5-kb insert encoding the 18S and 28S ribosomal units of *Drosophila melanogaster* (Meigen, 1830) (Endow 1982), and it was subsequently labelled with biotin-dUTP using a Nick Translation Kit (Abbott). In situ hybridisation of the probe with the chromosomal spreads was performed overnight according to a standard protocol and the probe signal was enhanced and detected using an avidin-FITC/biotinylated anti-avidin system (Vector Laboratories) at lower stringency according to previously described protocols [21,35]; in particular, the hybridisation mix consisted of 2.5 ng/μL of probe, 50% formamide, 10% dextran sulphate and 2xSSC, with an incubation time of 18 h at 37 °C. 

C-banding was completed with denaturation and formamide sequentially after FISH experiments according to the protocol of Fernandez et al. [36] and using CMA3 and DAPI staining counterstain. 

### 2.4. Microscopic Analysis and Imaging Processing

The metaphases were analysed under a Zeiss Axio2 epifluorescence microscope and captured using a coupled Zeiss digital camera. At least 10 metaphase spreads were analysed from each sample. 

## 3. Results

### 3.1. Karyotype Analysis

**Figure 1:**
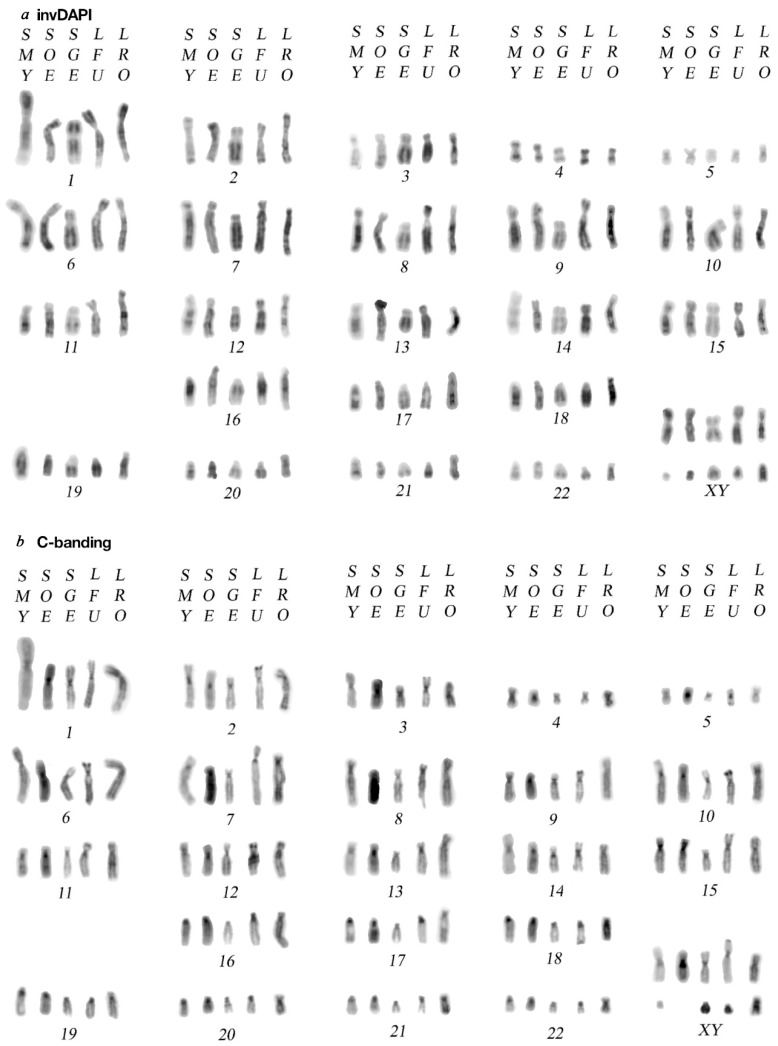
Comparative series of inverted DAPI banded (**a**) and C-banded chromosomes (**b**) of the analysed species: *Leontocebus fuscicollis, Leontopithecus rosalia, Saguinus geoffroyi, S. mystax* and *S. oedipus*.

**Figure 2:**
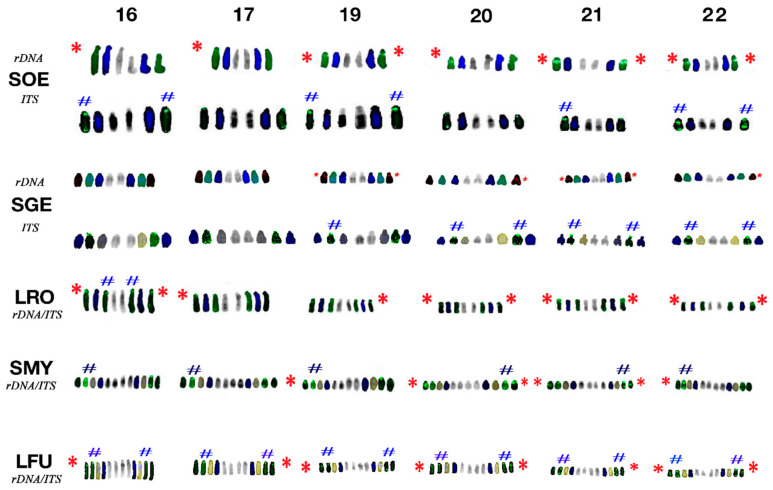
Chromosomes 16–17, 19–22 with rDNA and telomere loci mapping distribution for each species: *Saguinus oedipus, Saguinus geoffroy*, *Leontopithecus rosalia*, *Saguinus mystax*, *Leontocebus fuscicollis*. Each pair is reported covering the time when chromosomes were available after sequential DAPI, DAPI inverted, CMA3 staining and FISH mapping signals. Telomeric signals are in green, rDNA probes signals are in red except in SOE. The red stars label chromosomes with rDNA loci, the hash sign marks chromosomes with telomere probe amplification.

**Figure 3:**
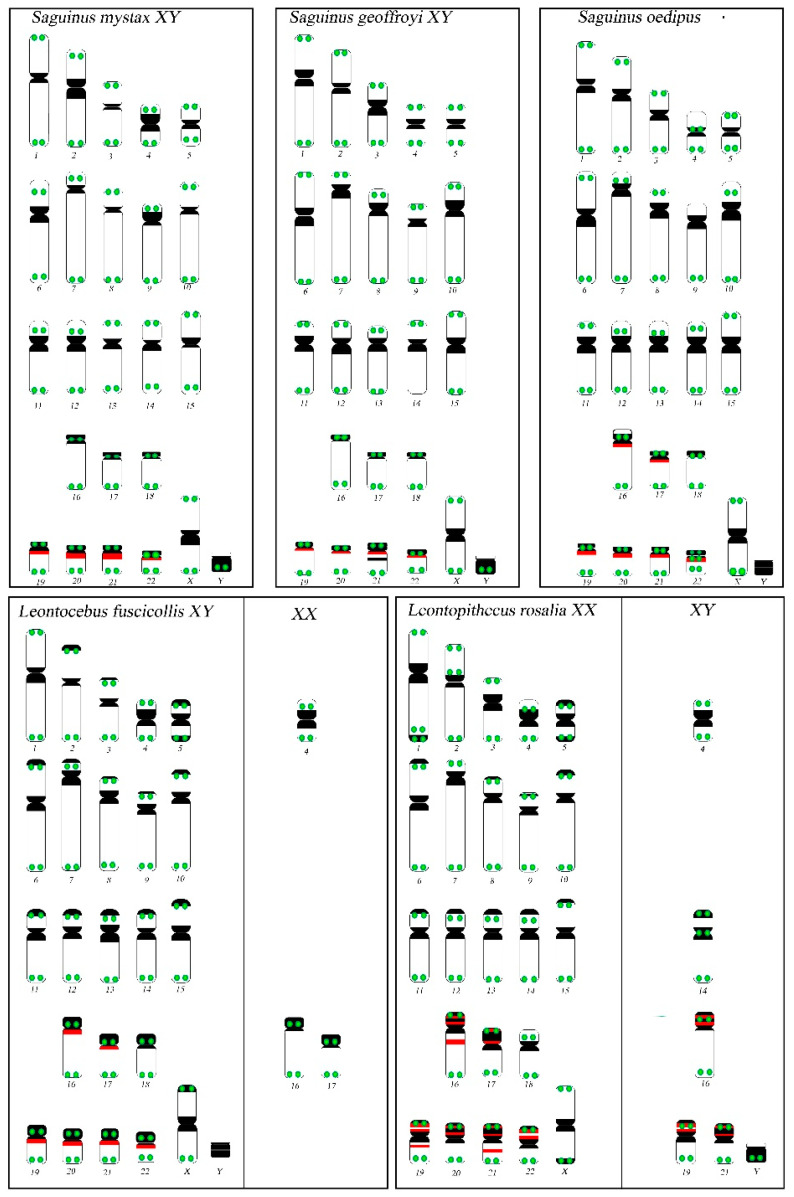
Patterns of the distribution of telomeric repeats, rDNA and heterocromatin in five tamarin species. Dark regions correspond to constitutive heterochromatin blocks. A schematic representation of chromosomes and bands was compiled from this study and others [9,11,29]. Telomeric signals are in green, rDNA probes signals are in red; chromosome variants are reported in the boxes on the right.

All analysed species shared the same diploid chromosome number: 2n = 46 (see Figure 1, Figure 2 and Figure 3, Figures S1–S5). In all individuals, the karyotype consisted of two metacentric pairs (4–5), 13 pairs of submetacentric chromosomes (1–3 and 6–15) and one pair of acrocentric chromosomes (18) (Figure 1a). Polymorphism between species was detected for the pairs 16–17, which are either acrocentric or subtelocentric, and for the subtelocentric pairs 19–22, which present differences in the length of the p arm, which is small in *Saguinus* and slightly bigger in *Leontocebus fuscicollis* and *Leontopithecus rosalia* (Figure 1a). The X chromosome has a similar size in all species. The Y chromosome is very acrocentric in *Leontopithecus rosalia, S. geoffroyi,* and *S. mystax*, while it is metacentric/submetacentric in *Leontocebus fuscicollis* and *Saguinus oedipus* (Figure 1a).

### 3.2. C-Banding and CMA3 Staining

C-banding data (Figure 1b) obtained in the present work agrees with literature [9,11]. In the species of the genus *Saguinus,* heterochromatin is mainly restricted to centromeric position in both biarmed and acrocentric chromosomes. Apart from centromeric signals, additional and peculiar C-positive bands were found on all chromosomes, especially at the distal p arms of submetacentric autosomes 2, 3, 6 and 8–15 in *Leontocebus fuscicollis* and *Leontopithecus rosalia*. Furthermore, on subtelocentric pairs 16–22 of *Leontocebus fuscicollis,* we could note a small p arm enriched in heterochromatin, while the homolog chromosomes in *Leontopithecus rosalia* showed additional accumulation of C-positive bands at the distal part of the p arms. CMA3 strongly stained regions rich in CG at centromeres where DAPI did not stain and it was useful to identify chromosomal regions enriched with repetitive elements (Figures S2–S4).

### 3.3. Fluorescence In Situ Hybridisation with an rDNA Probe

In the species of the genus *Saguinus*, rDNA loci were detected on the q arms of subtelocentric chromosomes 19–22 and additional rDNA loci were localised in chromosomes 16 and 17 (Figure 2, Figures S1–S5). The rDNA loci were detected on the subtelocentric chromosomes 19–22 on the q arm in *Leontocebus fuscicollis* and on the p arm in *Leontopithecus rosalia*, although with stronger accumulation in the latter (Figures S2–S4). Also, *Leontocebus fuscicollis* (male) and *Leontopithecus rosalia* showed additional rDNA signals on the subtelocentric chromosomes pairs 16 and 17 close to the centromere position. These signals however are not always present in all metaphases, presumably because of the low copy number of rDNA loci close to the limit of the detection threshold of the FISH method (Figures S4 and S5).

### 3.4. Fluorescence In Situ Hybridisation with (TTAGGG)n Probe

Telomeric motifs were detected at the ends of all chromosomes in all examined specimens. In the species of the genus *Saguinus* and in *Leontocebus fuscicollis,* peculiar and bright signals were detected on chromosomes 19–22, presumably as result of amplification process (Figure 2, Figures S1–S4). Furthermore, ITSs were at the centromeres of the subtelocentric chromosomes 16 and 17 in both male and female of *Leontocebus fuscicollis* and in *Saguinus oedipus* and on chromosomes 14 and 16 of the male *Leontopithecus rosalia.* In *Leontopithecus rosalia*, telomeric probe localisation differed between male and female individuals: indeed, in females, ITS was found on chromosomes 1, 2 and 4. Other peculiar signals were found on chromosome 6 p arm in *Saguinus mystax* and on chromosome 22 in *Saguinus oedipus* and in heterozygosis (Figure 1 and Figure S2). 

All the results are reported in a schematic representation (Figure 3).

## 4. Discussion

### 4.1. Karyotypic Variability in 2n = 46 Tamarins 

This study allowed us to explore the heterochromatin and repetitive sequence localisation in the genera *Leontocebus, Leontopithecus* and *Saguinus* in order to expand our understanding of chromosome evolution of these closely related New World primates. We analysed the localisation of telomeric sequences among tamarin species from the genus *Saguinus*, and also in the phylogenetically related *Leontocebus fuscicollis* and *Leontopithecus rosalia*. The inverted DAPI-banded karyotypes showed that all examined 2n = 46 species have similar karyotypes. A cross-species comparison of inverted DAPI-banded karyotypes is shown in Figure 1.

Our comparative analysis of inverted DAPI-banding reveals that the karyotypes of the genera *Saguinus* and *Leontocebus* mainly differ from *Leontopithecus* in the morphology of autosomal pairs 16, 17 and 19–22 (Figure 2). For example, chromosome pairs 16–17 are either acrocentric, as in *S. mystax* in agreement with the previous reconstructions in *S. midas* and *S. bicolor* where the homologs are identified as pairs 20–22 [11,13,29], or are subtelocentric, as in *Leontopithecus rosalia* (current study) and in *Leontopithecus chrysomelas* [14].

### 4.2. C-Banding Pattern Variation on Smaller Autosomes

Apart from classic bands at the centromeric position in all chromosomes, C-banding showed a slightly different pattern on pairs 16, 17 and 19-22 (Figure 1b). Indeed, on these chromosomes in the *Saguinus* species, we identified a positive C-band just below the centromere on the q arm; on the same homologs 16, 17 and 19–22 in *Leontocebus fuscicollis,* we found an enrichment of heterochromatin at the p arm and in *Leontopithecus* we found heterochromatin enrichment at the centromere and at the distal part of the p arms (Figure 1b and Figure 3). These results are in agreement with the previous comparison of the C-banding pattern performed on *Leontopithecus rosalia* and *Saguinus midas*, which showed differences in the variation, quantity and distribution of the non-centromeric constitutive heterochromatin [11,29]. Such a variable pattern of heterochromatin distribution often occurs among phylogenetically close species, even in other groups of mammals [22]. 

### 4.3. Topology of rDNA Loci

rDNA loci were mapped for the first time by FISH in *Leontocebus fuscicollis*, *Leontopithecus rosalia* and *S. mystax* in the current study. In contrast to a previous study, where classic silver stain permitted the detection of active NORs [11,29], we found both active and inactive rDNA loci in the species of the genus *Saguinus*. In particular, we detected rDNA probe signals on the p-arm of chromosome pairs 16 and 17 in *Leontopithecus rosalia* in agreement with a previous molecular evidence on *Leontopithecus chrysomelas* [14]. The rDNA signals on the q arm of pairs 16 and 17 have also been shown here for the first time in *Saguinus oedipus* and on respective homologs in the male *Leontocebus fuscicollis*, while previous analysis did not detect rDNA signals in *Saguinus oedipus* [11,22]. The different morphology of chromosome pairs 19–22 is also due to the fact that rDNA loci are found on the q arm in the genera *Leontocebus* and *Saguinus* and on the p arm in *Leontopithecus rosalia*, where we also show an extensive hybridisation signal (Figure 2 and Figure 3). This different location of rDNA signals could be explained by pericentric inversion dislocating the rDNA loci in the opposite arms from q arms in the former species to the p arms in *Leontopithecus* (Figure 3), in agreement with previous classic silver staining and molecular cytogenetic analysis applied respectively in several species of the genus *Saguinus* [7,11] and in *Leontopithecus chrysomelas* [14]. The extensive hybridisation signal on the p arm position in *Leontopithecus rosalia* also indicates that a paracentric inversion could have dislocated the rDNA loci. This evidence could be supported by the signal observed in *Leontopithecus chrysomelas* [14], where even if not underlined in that manuscript, the rDNA hybridisation pattern on 16, 17 and 19–22 pairs shows bright and amplified signals covering the whole p arm, presumably because of the extensive enrichment of rDNA loci. Moreover, we underline that the double rDNA pattern we show on chromosome pairs 19–22 in *Leontopithecus rosalia* overlaps with a strong C-positive block, which is especially evident when comparing the rDNA pattern with the previous C-banded karyotype [11]. Previous classic cytogenetic analyses led to the conclusion that the species *Leontopithecus rosalia* and *Saguinus midas* differ by at least four pericentric inversions [11], and our data analysis confirmed these intrachromosomal rearrangements at the molecular level in the species analysed in the present work.

### 4.4. Telomere Distribution

Apart from classic telomeric signals at the ends of chromosomes (Figure 3), we detected ITSs at the centromeres of the subtelocentric chromosomes 16 and 17 in both male and female *Leontocebus fuscicollis*, on chromosomes 16 of the male *Leontopithecus rosalia* and in *Saguinus oedipus*. The formation of ITSs could be explained due to either a very small pericentric inversion or an amplification of telomeric sequences and heterochromatin above the centromere (Figure 2 and Figure 3).

Moreover, bright telomeric signals were detected at centromeres on subtelocentric chromosomes pairs 19–22 in the genera *Saguinus* and *Leontocebus* (Figure 2 and Figure 3). Those signals are not present at the centromeres on the bigger subtelocentric homologs in the genus *Leontopithecus,* where only classic telomeric end signals were detected (Figure 2 and Figure 3). This different distribution might be the result of amplification of the terminal telomeric sequences in *Saguinus oedipus, S. mistax* and *S geoffroyi*, as well as of the split and loss of the signal intensity in *Leontopithecus rosalia*, presumably due to the inversions. 

Additional ITS signals were found in some autosomes in the different species such as for example on and 22 in *S. oedipu*s; however, these variable forms could be polymorphisms in tamarins worthy of further investigation.

## 5. Conclusions 

Despite the fact that the analysed species have the same diploid number and conservative karyotype morphology, differences were detected among chromosomal pairs 16, 17 and 19–22 due to variations in the accumulation of heterochromatin, rDNA loci and telomeric sequences and due to intrachromosomal rearrangements. The comparison of telomeric sequence signals and rDNA loci distribution between the genera *Saguinus*, *Leontocebus* and *Leontopithecus* allowed us to verify and confirm at the molecular level four pericentric inversions responsible for the differences between *Saguinu*s and *Leontopithecus* on chromosome pairs 19–22, which were previously proposed by classic cytogenetic methods, and that the comparative painting approach could not detect [35]. Moreover, mapping of rDNA loci revealed extensive enrichment on p arms of chromosomes pairs 19–22 in *Leontopithecus* as a result of paracentric inversions, presumably following the pericentric inversion, which are apomorphisms in relation to other tamarins. Furthermore, on pairs 16 and 17 in *Leontocebus fuscicollis Saguinus oedipus* and 16 in *Leontopithecus rosalia*, we also showed ITSs that could be due to an inversion and/or an amplification of heterochromatin above them and/or the accumulation of rDNA loci. Indeed, on those homologs in the other analysed species, the presence of these rDNA loci was also shown and are presumably present as polymorphisms. 

In general, our cytogenetic comparative analysis reveals differences in the karyotypes, especially between the genera *Saguinus/Leontocebus* and genus *Leontopithecus;* furthermore, the genera *Leontopithecus* and *Leontocebus* show a few apomorphic patterns, such as peculiar C-banding patterns. Considering that the small-bodied tamarins’ radiation has been understudied so far and that several species are endangered, we assumed that their cytogenetic features have been underestimated and additional species and populations should be studied in order to better understand their genome evolution and to clarify the role of repetitive sequences in the evolution and adaptive radiation of these derived platyrrhini species.

## Figures and Tables

**Table 1:** Information for all specimens analysed in the current study. ♂ male, ♀ famale.

Family	Latin Name	Sex ♂/♀	Cell Type	Acknowledgements
Cebidae	*Saguinus Oedipus* SOE	♀	fibroblast cell line	Melody Roelke (Frederick National Laboratory of Cancer Research, Leidos Biomedical Research, Frederick, MD, USA), June Bellizzi and Director Richard Hann (Catoctin Wildlife Park and Zoo, Thumont, MD, USA)
	*S. geoffroyi SGE*	♂		
	*S. mystax SMY*	♂/♀		
	*Leontocebus fuscicollis LFU*	♂/♀		
	*Leontopithecus rosalia LRO*	♂/♀		Dr. Stephen O’Brien, Mary Tompson (Laboratory of Genomic Diversity, National Cancer Institute, Frederick MD, USA), Dr. Mitchell Bush; National Zoological Park, Washington, DC

## Data Availability

All data are provided in the current manuscript.

## Supplementary Materials

The following are available online at https://www.mdpi.com/article/10.3390/biology10090844/s1, Figure S1. Reconstructed karyotype of *Saguinus oedipus* after sequential DAPI, DAPI inverted and FISH mapping (a), metaphases in DAPI inverted (b), metaphases in DAPI (c), rDNA probe mapping (d), telomeric probe mapping (e), a reconstructed karyotype of *S. oedipus* after sequential DAPI, DAPI inverted, FISH with telomeric probe (f).The red stars label NOR-bearing chromosomes, the hash sign marks chromosomes with telomeric signal amplification. Figure S2. Reconstructed karyotype of *Saguinus geoffroyi* after sequential DAPI, DAPI inverted, CMA3 staining and FISH mapping (a), metaphases in DAPI inverted (b), metaphases in DAPI (c), rDNA probe mapping (d), telomeric probe mapping (e), a reconstructed karyotype of *S. geoffroyi* after sequential DAPI, DAPI inverted, CMA3 staining and FISH with telomeric probe (f). The red stars label NOR-bearing chromosomes, the hash sign marks chromosomes with telomeric signal amplification. Figure S3. *Leontopithecus rosalia* metaphases in DAPI inverted (a), in DAPI (b), rDNA probe mapping (c), telomeric probe mapping (d); the reconstructed karyotype of *L. rosalia* after sequential CMA3/ DAPI, DAPI inverted, FISH with Telomeric and rDNA probes. The red stars label NOR-bearing chromosomes, the hash sign marks chromosomes with telomeric signal amplification. Figure S4. *Saguinus mystax* metaphases in DAPI inverted (a), in DAPI (b), rDNA probe mapping (c), telomeric probe mapping (d); the reconstructed karyotype of *S. mystax* after sequential CMA3/DAPI, DAPI inverted, FISH with telomeric and rDNA probes, and C banding obtained after chromosome denaturation in formamide (f). The red stars label NOR-bearing chromosomes, the hash sign marks chromosomes with telomeric signal amplification. Figure S5. *Leontocebus fuscicollis* metaphases in DAPI inverted (a), DAPI (b), rDNA probe mapping (c), telomeric probe mapping (d). The karyotype of *L. fuscicollis* after sequential CMA3/DAPI staining, FISH with telomeric and rDNA probes, and C banding obtained after chromosome denaturation in formamide. The red stars label NOR-bearing chromosomes, the hash sign marks chromosomes with telomeric signal amplication.
