# Supplementary material for: Repetitive Sequence Distribution on Saguinus, Leontocebus and Leontopithecus Tamarins (Platyrrhine, Primates) by Mapping Telomeric (TTAGGG) Motifs and rDNA Loci
Source: Biology (Basel). 2021 Aug 30;10(9):844. doi: 10.3390/biology10090844 (PMC8470041; doi:10.3390/biology10090844)

Saguinus oedipus

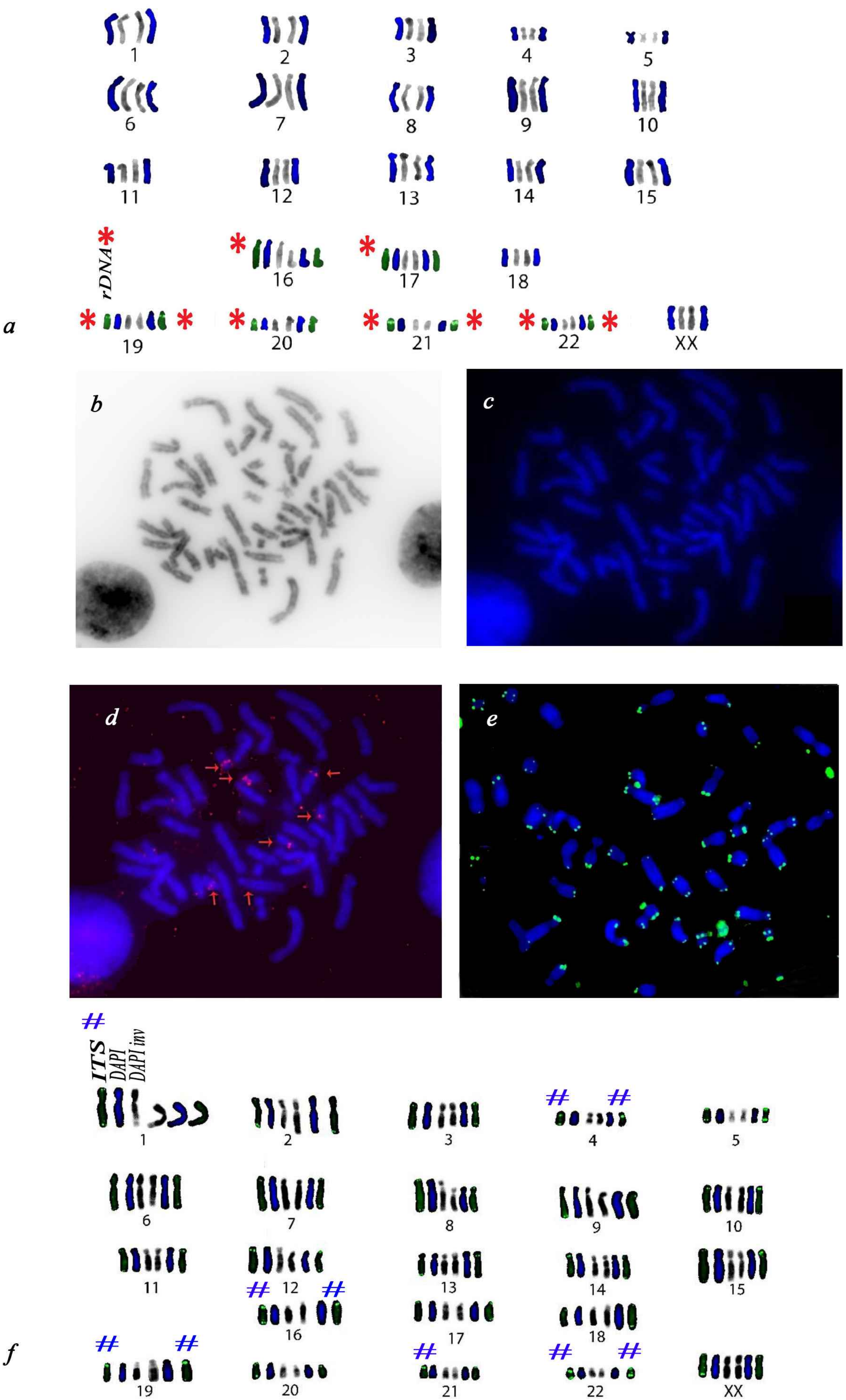

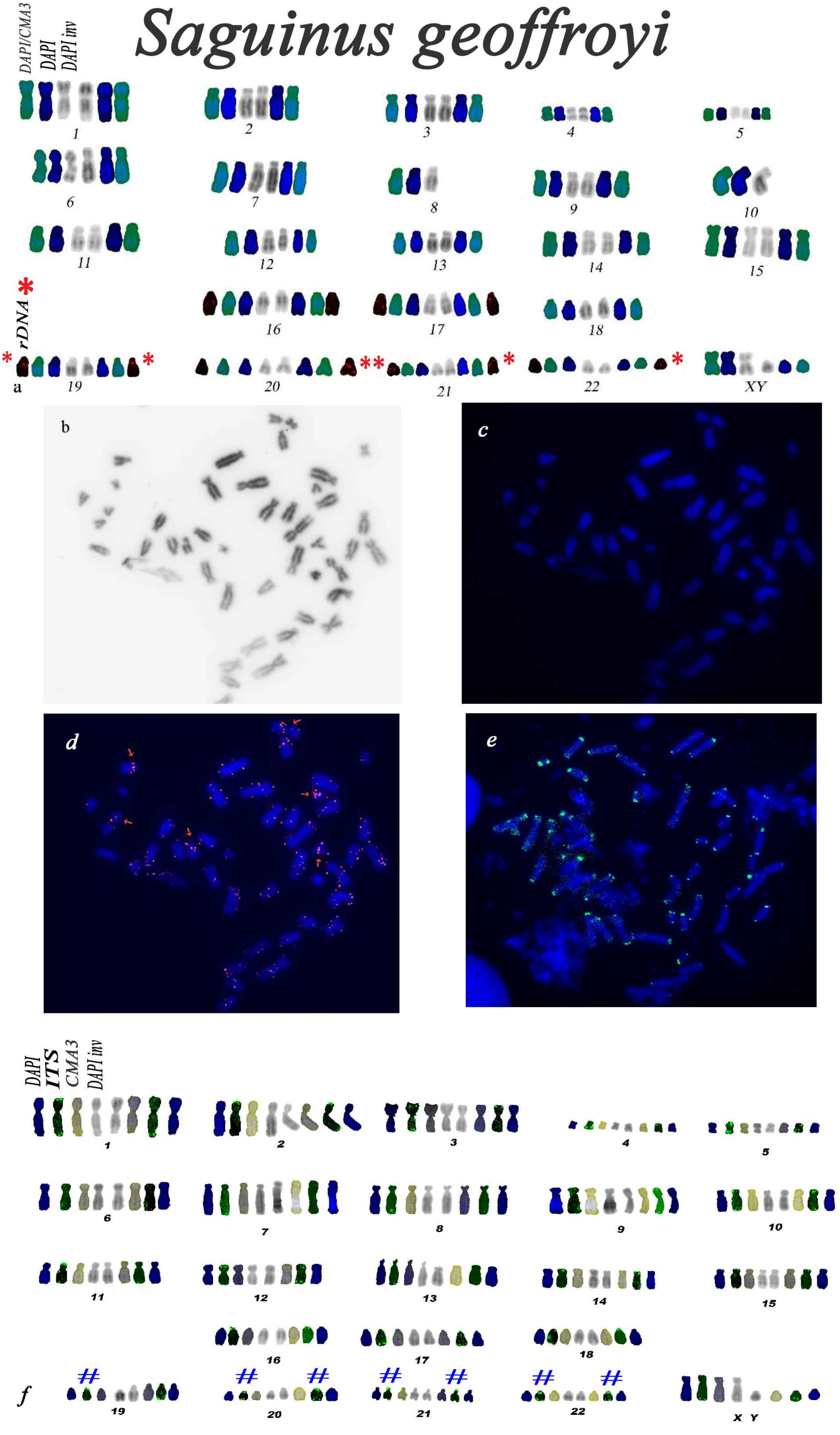
Saguinus geoffroyi
DAPI/CMA3
DAPI
DAPI inv
rDNA
a
b
c
d
e
f
DAPI
ITS
CMA3
DAPI inv
1 2 3 4 5 6 7 8 9 10 11 12 13 14 15 16 17 18 19 20 21 22 XY

Leontopithecus rosalia

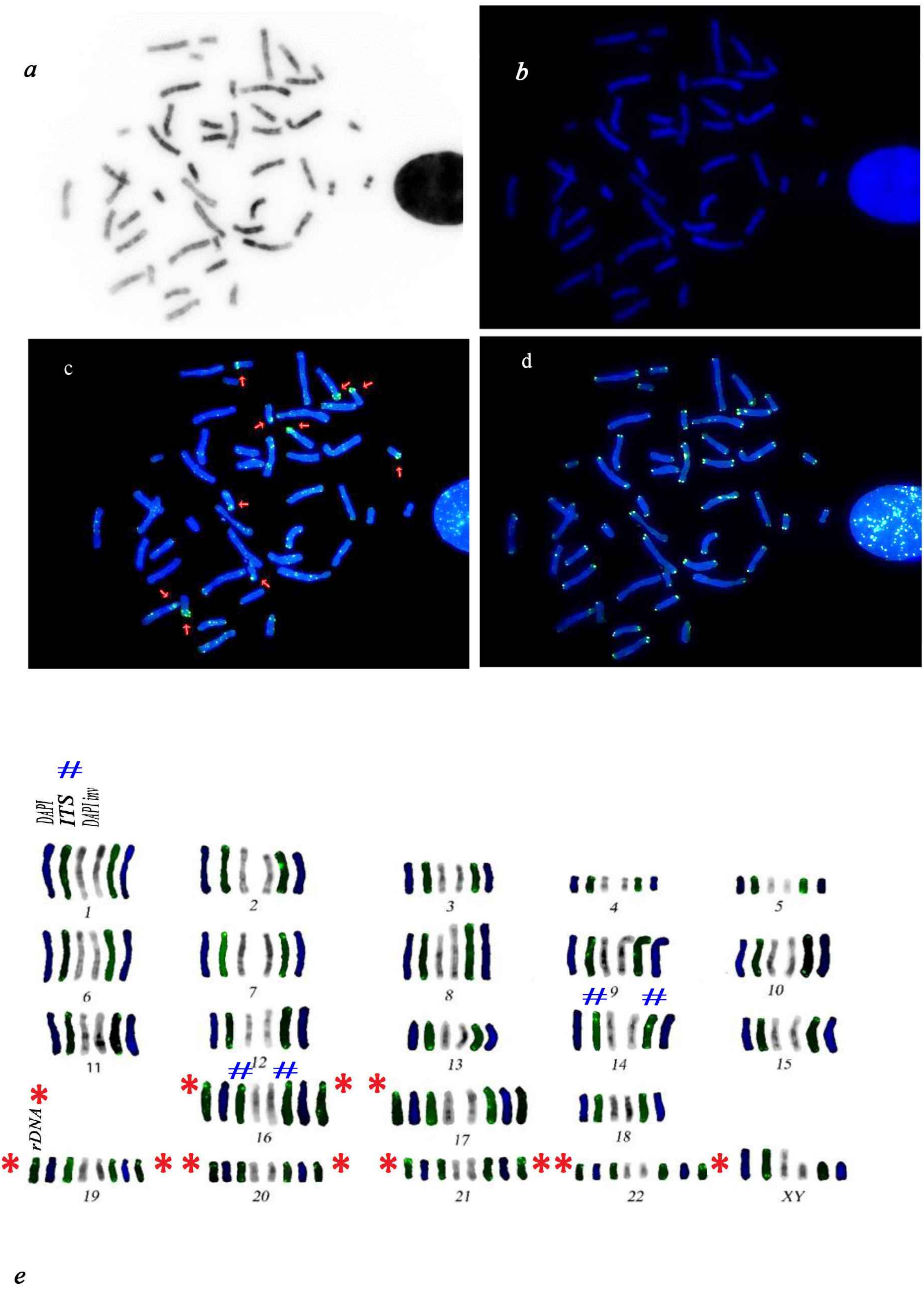

Saguinus mystax

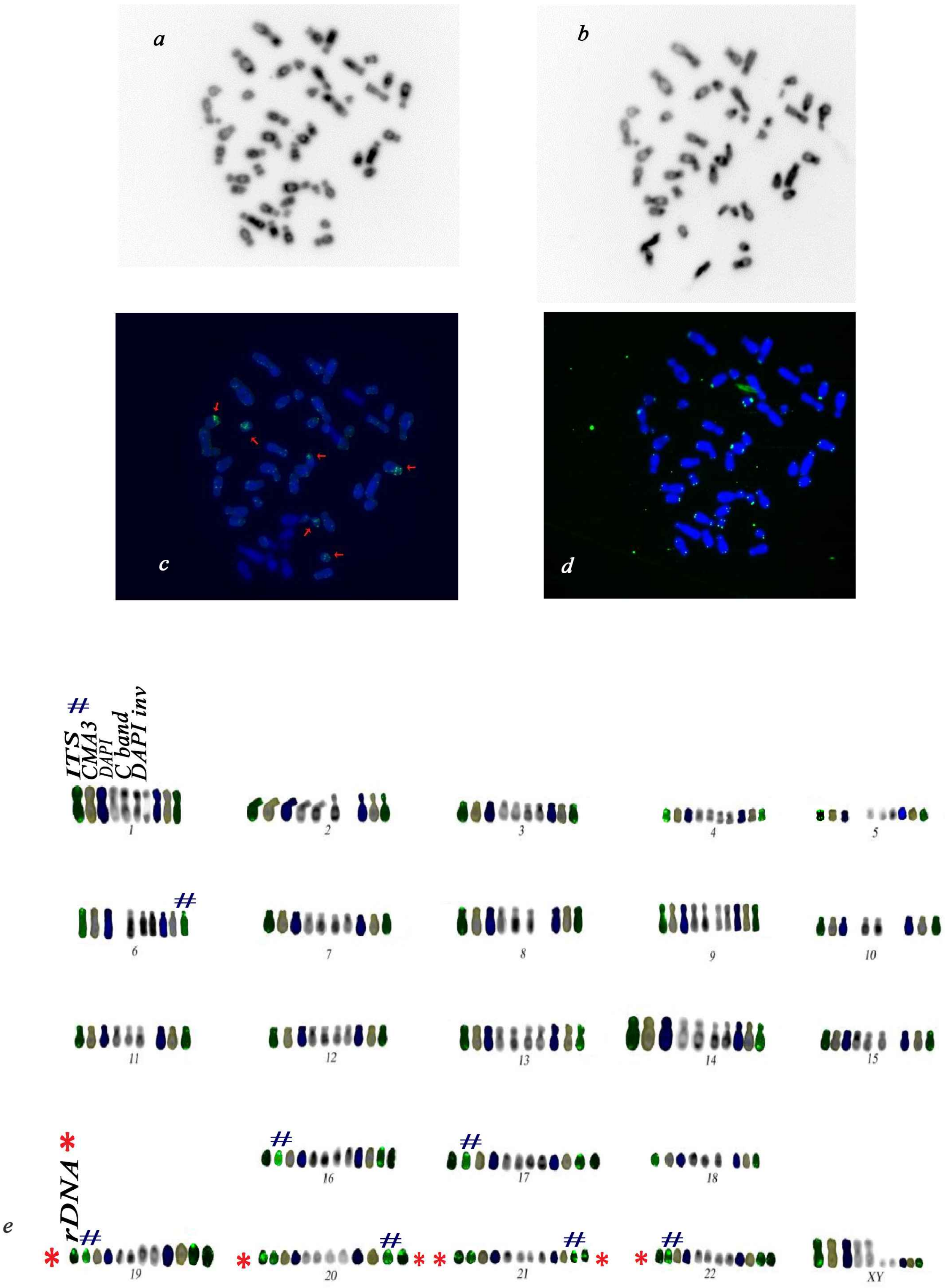

Leontocebus fuscicollis

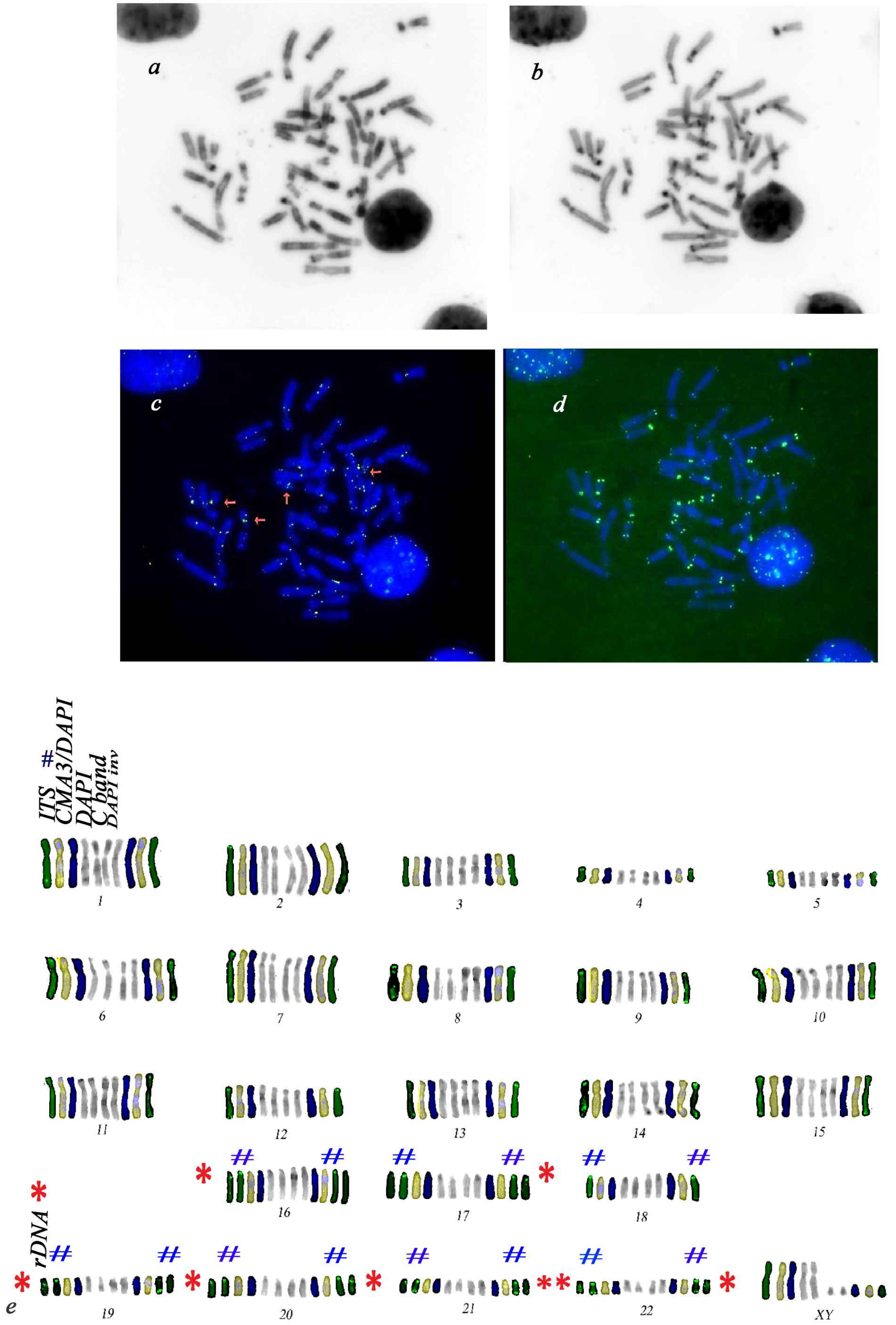

Supplement: Supplementary file 1 [file biology-10-00844-s001.zip › biology-1294410-sup.pdf]
